# The classical hitchhiking model with continuous mutational pressure and purifying selection

**DOI:** 10.1002/ece3.8259

**Published:** 2021-10-27

**Authors:** Wolfgang Stephan

**Affiliations:** ^1^ Leibniz‐Institute for Evolution and Biodiversity Science Natural History Museum Berlin Germany

**Keywords:** genetic hitchhiking, mutation pressure, purifying selection, selective sweeps, soft sweeps

## Abstract

Detecting selective sweeps driven by strong positive selection and localizing the targets of selection in the genome play a major role in modern population genetics and genomics. Most of these analyses are based on the classical model of genetic hitchhiking proposed by Maynard Smith and Haigh (1974, *Genetical Research*, 23, 23). Here, we consider extensions of the classical two‐locus model. Introducing mutation at the strongly selected site, we analyze the conditions under which soft sweeps may arise. We identify a new parameter (the ratio of the beneficial mutation rate to the selection coefficient) that characterizes the occurrence of multiple‐origin soft sweeps. Furthermore, we quantify the hitchhiking effect when the polymorphism at the linked locus is not neutral but maintained in a mutation‐selection balance. In this case, we find a smaller relative reduction of heterozygosity at the linked site than for a neutral polymorphism. In our analysis, we use a semi‐deterministic approach; i.e., we analyze the frequency process of the beneficial allele in an infinitely large population when its frequency is above a certain threshold; however, for very small frequencies in the initial phase after the onset of selection we rely on diffusion theory.

## Abstract 2

We consider some extensions of the classical two‐locus hitchhiking model. Introducing mutation at the strongly selected site, we analyze the conditions under which soft sweeps may arise. Furthermore, we quantify the hitchhiking effect when the polymorphism at the linked locus is not neutral but maintained in a mutation‐selection balance.

## 1 INTRODUCTION

When a selectively favored mutation occurs in a population and is subsequently fixed, it is inevitable that the frequency of linked neutral variants will be altered. In a seminal paper, Maynard Smith and Haigh (1974) described this process and termed it *genetic hitchhiking*. They showed that in large populations a single hitchhiking event may temporarily reduce neutral genetic variation around the site of selection. In recombining organisms the size of the region of reduced variation depends critically on the ratio of the recombination rate and the selection coefficient of the favorable mutation and may be limited to a relatively small fraction of the genome. In nonrecombining organisms such as bacteria, however, variation on entire chromosomes may be eliminated by genetic hitchhiking.

The hitchhiking model was revisited in the late 1980s to describe patterns of reduced variation in DNA polymorphism data, which were found in genomic regions of low recombination rates around centromeres and telomeres of *Drosophila* (Aguade et al., 1989; Begun & Aquadro, 1992; Stephan & Langley, 1989) and also on the fourth chromosome (Berry et al., 1991). Begun and Aquadro further showed that levels of DNA variation correlate with recombination rates across much of the *Drosophila melanogaster* genome, whereas average divergence to its sibling species *Drosophila simulans* was hardly affected by recombination. Given these data, the deterministic hitchhiking model of Maynard Smith and Haigh (1974) was extended by Kaplan et al. (1989) who analyzed a stochastic version of the process (including genetic drift) by means of coalescent theory. Furthermore, Stephan et al. (1992) studied genetic hitchhiking using the diffusion equation method. Alternative approximations of the hitchhiking model were provided by Barton (1998) and Gillespie (2000).

In population genetics, the concept of “genetic hitchhiking” is now more broadly used than around the year 1990 and describes any situation in which changes in allele frequencies caused by relatively strong selection affect the frequencies of neutral or weakly selected variants at linked sites in the genome. This includes—for instance—the case of balancing selection (Kaplan et al., 1988) and also background selection (Charlesworth et al., 1993). At the same time, and more specifically, for genetic hitchhiking caused by positive directional selection (as considered by Maynard Smith and Haigh), the term *selective sweep* is now generally used, which was introduced by Berry et al. (1991).

Several controversies have surrounded the application of the selective sweep model to data. Charlesworth et al. (1993) have explained the observed reduction of nucleotide variation in genomic regions of reduced recombination rates by background selection. According to this model, the level of neutral (or nearly neutral) variation can be reduced below classical neutral expectation by selection against the steady input of deleterious mutations. Furthermore, it has been difficult to distinguish the effect of selective sweeps from that of specific demographic scenarios, in particular bottlenecks (Pavlidis et al., 2010). Another controversy arose between selective sweeps and so‐called soft sweeps (Jensen, 2014). The latter may be caused by positive directional selection on standing genetic variation after an environmental change or by multiple beneficial mutations segregating simultaneously in a population (Hermisson & Pennings, 2005, 2017; Innan & Kim, 2004). Despite substantial efforts from many theorists and empiricists, fundamental questions on the relationship of demography, selective sweeps, soft sweeps, and background selection with regard to data analysis are still open. However, since these issues are not a focus of this study, the reader is referred to the work of Li and Stephan (2006), Elyashiv et al. (2016), Comeron (2017), Harris et al. (2018), Garud et al. (2021), or to the Perspectives article by Stephan (2019).

This article is devoted almost exclusively to the modeling efforts of selective sweeps by extending the classical hitchhiking model. We begin by formulating the model of Maynard Smith and Haigh (1974) more generally as a two‐locus two‐allele model with additive fitness. Besides strong positive directional selection at the selected locus, we allow for weak purifying selection at the linked locus. Furthermore, we introduce mutation at both loci. This allows us to address the following topics: First, following Maynard Smith and Haigh we perform a deterministic analysis of the extended hitchhiking model. This analysis is valid after the trajectory of the strongly advantageous allele has reached a certain threshold frequency. Second, we derive analytical results that show under which conditions soft sweeps caused by multiple beneficial mutations segregating in a population (so‐called multiple‐origin soft sweeps) are predicted by our extended hitchhiking model and identify a new parameter characterizing the occurrence of this type of soft sweeps. Third, we quantify the hitchhiking effect (i.e., the degree of reduction of variation) under the assumption that the polymorphism at the linked locus is not neutral but in a mutation‐selection balance. Fourth, we analyze the initial phase of the frequency process of strongly beneficial alleles after the onset of positive selection (until it reaches $x_{0}$) by diffusion theory. This allows us to derive initial conditions for our deterministic analyses mentioned above.

## 2 DETERMINISTIC HITCHHIKING MODEL

To extend the classical hitchhiking model (Maynard Smith & Haigh, 1974), it is convenient to start from a diploid, two‐locus two‐allele model with additive fitness (Bürger, 2000, Chapter II.1). In this model, selection at both loci may be introduced in a straightforward way as well as mutation and recombination between both loci. Calling the alleles at the first locus *A* and *a*, where *A* is the major allele, and those at the second locus *B* and *b*, we denote the possible gametes as *AB*, *aB*, *Ab*, and *ab*, and the relative frequencies of these gametes are $x_{1},x_{2},x_{3}$, and $x_{4}$. They add up to 1. Wiehe (1995, Chapter 4) derived equations for this model including viability selection and two‐way mutation at both loci and recombination between loci. The ordinary differential equations (ODEs) of this model are as follows.

(1) $${\dot{x}}_{1}=‐\left({\bar{\mu }}_{A}+{\bar{\mu }}_{B}\right)x_{1}+\mu_{A}x_{2}+\mu_{B}x_{3}+s_{1}x_{1}\left(1‐x_{1}‐x_{3}\right)+s_{2}x_{1}\left(1‐x_{1}‐x_{2}\right)‐rD$$

(2) $${\dot{x}}_{2}=‐\left(\mu_{A}+{\bar{\mu }}_{B}\right)x_{2}+{\bar{\mu }}_{A}x_{1}+\mu_{B}x_{4}‐s_{1}x_{2}\left(x_{1}+x_{3}\right)+s_{2}x_{2}\left(1‐x_{1}‐x_{2}\right)+rD$$

(3) $${\dot{x}}_{3}=‐\left({\bar{\mu }}_{A}+\mu_{B}\right)x_{3}+\mu_{A}x_{4}+{\bar{\mu }}_{B}x_{1}+s_{1}x_{3}\left(1‐x_{1}‐x_{3}\right)‐s_{2}x_{3}\left(x_{1}+x_{2}\right)+rD,$$

where a dot denotes differentiation with respect to time. $\mu_{A}$ is the mutation rate from allele *a* to *A*, and ${\bar{\mu }}_{A}$ that in the opposite direction. Similarly, $\mu_{B}$ denotes the mutation rate from *b* to *B*. To maintain the property of the original model that the positively selected mutation at the second locus gets fixed at the end of a sweep, we put ${\bar{\mu }}_{B}=0$. The selection coefficients at the first and second locus are given by $s_{1}$ and $s_{2}$, respectively. We assume that the absolute value of $s_{1}$ is generally (much) smaller than $s_{2}$, which is positive and characterizes the fitness advantage of the beneficial allele. The recombination fraction between the two loci is *r*, and $D=x_{1}x_{4}‐x_{2}x_{3}$ measures linkage disequilibrium (LD).

The model described by the above equations is different from the model proposed by Maynard Smith and Haigh (1974) as it allows for mutation at both loci and variation at the first locus may deviate from neutrality. We will explore next to what extent this more general model can be treated analytically. Subsequently, because the deterministic model is not valid for very small frequencies of the beneficial allele (Kaplan et al., 1989), we analyze the initial phase of the adaptive process stochastically. This allows us to specify the state of the above variables at time $t_{0}$ at which the deterministic phase begins.

## 3 ANALYSIS OF THE DETERMINISTIC PHASE

Following Maynard Smith and Haigh (1974), we introduce the coordinates $p_{1}$, the frequency of *A* alleles in chromosomes containing *B*, and $p_{2}$, the frequency of *A* in *b*‐chromosomes. Thus, assuming x is the frequency of the selected allele *B*, we have $p_{1}=\frac{x_{1}}{x}$ and $p_{2}=\frac{x_{3}}{1‐x}$. A consequence of this variable change is that we can analyze the model only in the interval 0<x<1. As explained above, this is not a severe limitation as our deterministic treatment is not valid very close to the boundary 0 anyway (Kaplan et al., 1989). With this transformation of variables, the ODEs (1)–(3) become

(4) $$\dot{x}=\left\{\left[s_{1}\left(p_{1}‐p_{2}\right)+s_{2}\right]x+\mu_{B}\right\}\left(1‐x\right)$$

(5) $${\dot{p}}_{1}=‐{\bar{\mu }}_{A}p_{1}+\mu_{A}\left(1‐p_{1}\right)+s_{1}p_{1}\left(1‐p_{1}\right)‐r\left(1‐x\right)\left(p_{1}‐p_{2}\right)‐\mu_{B}\frac{1‐x}{x}\left(p_{1}‐p_{2}\right)$$

(6) $${\dot{p}}_{2}=‐{\bar{\mu }}_{A}p_{2}+\mu_{A}\left(1‐p_{2}\right)+s_{1}p_{2}\left(1‐p_{2}\right)+rx\left(p_{1}‐p_{2}\right).$$

Eq. (4) results from adding ODEs (1) and (2) and putting $x=x_{1}+x_{2}$. Eqs. (5) and (6) exploit the equality

(7) $$D=x\left(1‐x\right)\left(p_{1}‐p_{2}\right).$$

Eq. (4) indicates that the beneficial allele *B* may be driven by three forces: positive directional selection at the second locus, mutation at the second locus, and selection at the first locus (via LD between the first and second locus; see Eq. (7)).

In the following, we analyze the behavior of the deterministic model in several distinct parameter ranges. In each case, strongly positive directional selection at the second locus is assumed to be present.

Case 1: $\mu_{B}>0,r=0,\mu_{A}={\bar{\mu }}_{A}=0,s_{1}=0$.

It is informative to first consider the effect of mutation and strong directional selection at the second locus on neutral variation (at the first locus) alone. Using the assumption that all parameter values are zero, with the exception of $\mu_{B},s_{2}>0,$ it follows from Eqs. (4)–(6) that

(8) $$\dot{x}=\left(s_{2}x+\mu_{B}\right)\left(1‐x\right)$$

and

(9) $${\dot{p}}_{1}‐{\dot{p}}_{2}=‐\mu_{B}\frac{1‐x}{x}\left(p_{1}‐p_{2}\right).$$

These ODEs can be easily integrated by dividing Eq. (9) by Eq. (8). This leads to

(10) $$\frac{d\left(p_{1}‐p_{2}\right)}{\mathrm{dx}}=‐\mu_{B}\frac{p_{1}‐p_{2}}{x\left(s_{2}x+\mu_{B}\right)}.$$

Separation of variables then yields

(11) $$p_{1}‐p_{2}=C\frac{s_{2}x+\mu_{B}}{x},$$

where the integration constant is given as

(12) $$C=\frac{x_{0}}{s_{2}x_{0}+\mu_{B}}\left(p_{10}‐p_{20}\right).$$

The initial values (at $t=t_{0})$ of the variables $x,p_{1}$, and $p_{2}$ are denoted by the index 0. Simulations by Kaplan et al. (1989) suggest that $x_{0}$ should be at least as high as $\frac{5}{\alpha }$, where α is given by $2Ns_{2}$ in diploid populations of size N.

Eqs. (11) and (12) can be used to calculate the allele frequencies $x_{1}$ and $x_{2}$ as a function of x (or alternatively as a function of t by solving Eq. (8)). From Eq. (6) follows that $p_{2}$ does not depend on mutation at the second locus. Therefore,

(13) $$p_{2}=p_{20}.$$

The allele frequencies *AB* and *aB* are then given as

(14) $$x_{1}=C\left(s_{2}x+\mu_{B}\right)+p_{20}x$$

and

(15) $$x_{2}=x‐x_{1},$$

respectively.

Based on these results, we can address the question whether multiple‐origin soft sweeps (Hermisson & Pennings, 2005, 2017) are predicted by our extended hitchhiking model. In our model, such a soft sweep occurs if both gametes *AB* and *aB* are simultaneously present in a population in substantial amounts. Eq. (8) suggests that the probability of a sweep being soft is significantly high if the mutation rate $\mu_{B}$ is sufficiently large compared to the selection pressure $s_{2}x$ (Johri et al., 2021); i.e.,

(16) $$\mu_{B}\geq s_{2}x.$$

Of course, this inequality holds only for very small values of x. It describes the situation in which initially the frequency of the beneficial allele does not increase in an exponential fashion (as in the case of the classical hitchhiking model in which a single beneficial allele is assumed to be present at the onset of selection). Instead, in our case this exponential increase is delayed until the process reaches values of $x>\frac{\mu_{B}}{s_{2}}$. This suggests that $\frac{\mu_{B}}{s_{2}}$ represents a threshold parameter such that for $x<\frac{\mu_{B}}{s_{2}}$ mutation dominates selection, whereas for $x>\frac{\mu_{B}}{s_{2}}$ the opposite occurs. More multiple‐origin soft sweeps should arise with increasing values of $\frac{\mu_{B}}{s_{2}}$ for the following reason: A larger mutation rate increases the frequency of B alleles and a larger selection strength reduces their fixation time. Therefore, if a first mutation that arose on an *A*‐chromosome is on its way to fixation, the probability that a second beneficial mutation arises on an *a*‐chromosome and substantially increases in frequency becomes smaller.

Note that we have $\frac{\mu_{B}}{s_{2}}<x_{0}=\frac{5}{\alpha }$ for realistic values of population size and beneficial nucleotide mutation rate. Therefore, $\frac{\mu_{B}}{s_{2}}$ likely falls into the interval in which a stochastic treatment of the x process is required. Nonetheless, the above argument that is derived from Eq. (8) holds. The reason is that the right‐hand side of Eq. (8) is identical to the drift coefficient of the diffusion equation (except for the scaling factor 2N; see Eqs. (39) and (41) below).

Our analysis adds a new piece to the theory of Hermisson and Pennings (2005, 2017) who studied the occurrence of soft sweeps in a population of finite size. In their approach, the probability for mutation‐based soft sweeps largely depends on a single parameter Θ, which is a scaled beneficial mutation rate that accounts for many short‐term processes going on in a population (see the detailed discussion of short‐term effective population size and the target size of beneficial mutations in Hermisson and Pennings (2017)).

Very recently, Feder et al. (2021) reported simulations of a model that is virtually identical to the hitchhiking model described here, except that it is haploid and consists of only a single locus at which beneficial mutations were allowed to arise such that each mutation created a new allele. In their simulations, mutation rate μ was fixed, while the selection coefficient s and population size N varied as did θ=Nμ. For many parameter combinations, they ran forward simulations and recorded the percentage of runs in which the sum the frequencies of all mutations reached 50% by generation 30. In this case, a run was counted as a sweep. If a sweep occurred, they also checked whether more than one allele was at frequency >5%, which was counted as a multiple‐origin soft sweep. In their Figure 3C, they show that indeed for all values of s increasing θ led to a higher percentage of soft sweeps, whereas Ns had almost no effect. These observations are consistent with the theory of Hermisson and Pennings (2017). A strong effect was also found for selection. Increasing s led to a remarkable reduction of the percentage of soft sweeps, which is in qualitative agreement with our analysis.

Finally, we discuss the role of $p_{10}$ and $p_{20}$ in the detection of soft sweeps. As we show in the stochastic analysis below, $p_{20}$ is approximately given by the frequency $x_{3}^{*}$ of the major allele *A* at the onset of selection, while $p_{10}$ may be small due to the relatively large variance of x and $x_{1}$ in the initial phase. For $p_{10}<\varepsilon$, where ε is the detection threshold of a soft sweep conditional on a sweep is occurring, *AB* gametes may remain undetected, if the mutation rate is too small (see Appendix A).

Case 2: $\mu_{B}>0,r\geq 0,\mu_{A}\geq 0,{\bar{\mu }}_{A}\geq 0,s_{1}=0$.

We first analyze the joint effects of mutation at the second locus and recombination between both loci. Eqs. (9) and (10) then become

(17) $${\dot{p}}_{1}‐{\dot{p}}_{2}=‐r\left(p_{1}‐p_{2}\right)‐\mu_{B}\frac{1‐x}{x}\left(p_{1}‐p_{2}\right)$$

and

(18) $$\frac{d\left(p_{1}‐p_{2}\right)}{\mathrm{dx}}=‐r\frac{p_{1}‐p_{2}}{\left(1‐x\right)\left(s_{2}x+\mu_{B}\right)}‐\mu_{B}\frac{p_{1}‐p_{2}}{x\left(s_{2}x+\mu_{B}\right)}.$$

The latter ODE may be integrated in a similar way as Eq. (10) such that

(19) $$p_{1}‐p_{2}=\tilde{C}\frac{{\left(1‐x\right)}^{\rho }{\left(s_{2}x+\mu_{B}\right)}^{1‐\rho }}{x},$$

where $\rho =\frac{r}{s_{2}+\mu_{B}}$ and the integration constant is

(20) $$\tilde{C}=\frac{x_{0}}{{\left(1‐x_{0}\right)}^{\rho }{\left(s_{2}x_{0}+\mu_{B}\right)}^{1‐\rho }}\left(p_{10}‐p_{20}\right).$$

Next, we calculate $p_{2}$ using ODE (6). Inserting Eq. (19) into ODE (6) yields

(21) $${\dot{p}}_{2}=r\tilde{C}{\left(1‐x\right)}^{\rho }{\left(s_{2}x+\mu_{B}\right)}^{1‐\rho }.$$

This equation can be integrated taking into account that ρ≪1 and dividing Eq. (21) by Eq. (8). This leads to

(22) $$p_{2}\approx p_{20}‐r\tilde{C}\ln\left(1‐x\right).$$

Since ρ≪1, which appears to be biologically realistic, Eq. (19) is nearly identical to Eq. (11) and Eq. (22) is very similar to Eq. (13). Therefore, in the presence of strong selection and mutation at the second locus recombination has only a very weak effect on the dynamics of the frequencies of the *AB* and *aB* gametes. This may be surprising, given the distinct effect of recombination in the presence of strong selection on heterozygosity at the neutral locus in the study of Maynard Smith and Haigh (1974). The critical difference between our model and the original one by Maynard Smith and Haigh, however, is mutation. Without mutation at the selected locus, our approach would lead to the same predictions as that of Maynard Smith and Haigh. In other words, the presence of mutation alters the typical sweep (hitchhiking) effect of the Maynard Smith–Haigh model by generating more than one haplotype with a selected allele in the initial phase that may lead to partial parallel sweeps.

Finally, we discuss the effect of mutation at the first locus in conjunction with recombination, mutation, and selection at the second locus. In this case, the difference between ODEs (5) and (6) is formally identical to Eq. (17) and can be integrated as shown above in Eqs. (18)–(20), assuming that the selection coefficient is much larger than the mutation rates at the first locus and r. In a similar way as above, $p_{2}$ can be calculated.

Case 3: $\mu_{B}=0,r\geq 0,\mu_{A}>0,s_{1}<0$.

Here, we analyze the case studied by Maynard Smith and Haigh (1974), except that the polymorphism at the first locus is not neutral, but allele *A* is deleterious (maintained in a mutation‐selection balance). Thus, in this subsection *A* is not the major allele. From Eqs. (4)–(6), we get the following ODEs

(23) $$\dot{x}=\left[s_{1}\left(p_{1}‐p_{2}\right)+s_{2}\right]x\left(1‐x\right)$$

(24) $${\dot{p}}_{1}=\mu_{A}\left(1‐p_{1}\right)+s_{1}p_{1}\left(1‐p_{1}\right)‐r\left(1‐x\right)\left(p_{1}‐p_{2}\right)$$

(25) $${\dot{p}}_{2}=\mu_{A}\left(1‐p_{2}\right)+s_{1}p_{2}\left(1‐p_{2}\right)+rx\left(p_{1}‐p_{2}\right).$$

The frequency of *A* in the mutation‐selection balance at the first locus is given by $x_{30}=\frac{\mu_{A}}{\left|s_{1}\right|}.$ Since the frequency of *A* is assumed to be small, reverse mutation from *A* to *a* is neglected.

A general analytical solution of this system of ODEs is difficult to obtain, but we may approximate these equations under the assumption that the frequency $x_{30}$ of the deleterious allele *A* is relatively small such that a strongly advantageous mutation occurring at the second locus at t=0 hits a chromosome carrying allele *a* with high probability. In other words, we consider the following initial conditions at $t=t_{0}$

(26) $$p_{10}=0$$

(27) $$p_{20}=\frac{x_{30}}{1‐x_{0}}\approx x_{30}.$$

Furthermore, we assume that both r and $\left|s_{1}\right|\ll s_{2}.$ Under these assumptions, the quantities $p_{1}$ and $p_{2}$ remain small (compared to 1), while the strongly selected allele *B* increases from $x_{0}$ to $1‐x_{0}$ (i.e., near fixation). Then, from ODEs (23)–(25) we obtain the following equations

(28) $$\dot{x}\approx s_{2}x\left(1‐x\right)$$

(29) $${\dot{p}}_{1}‐{\dot{p}}_{2}\approx ‐\mu_{A}\left(p_{1}‐p_{2}\right)+s_{1}\left(p_{1}‐p_{2}\right)‐r\left(p_{1}‐p_{2}\right)$$

(30) $${\dot{p}}_{1}\approx s_{1}p_{1}+\mu_{A}‐r\left(1‐x\right)\left(p_{1}‐p_{2}\right).$$

Equations (28) and (29) can be readily integrated using the initial conditions (26) and (27) and expressing time t by $\tau =t‐t_{0}.$ Inserting the solutions into ODE (30) leads to a linear ODE of first order that can be solved. The resulting equation for $p_{1}$ contains an integral over a function that can be approximated by replacing the denominator of this function by $e^{‐s_{2}\tau^{\prime }}$ (see eqs. (14b) and (14c) of Stephan et al. (1992)). Thus, we have

(31) $$x\approx \frac{x_{0}}{x_{0}+\left(1‐x_{0}\right)e^{‐s_{2}\tau }}$$

(32) $$p_{1}‐p_{2}\approx ‐p_{20}e^{\left(s_{1}‐\mu_{A}‐r\right)\tau }$$

(33) $$p_{1}\approx p_{20}\left(1‐e^{s_{1}\tau }\right)+p_{20}\frac{r}{\mu_{A}+r}e^{s_{1}\tau }\left(1‐e^{‐\left(\mu_{A}+r\right)\tau }\right)$$

(34) $$p_{2}=p_{1}‐\left(p_{1}‐p_{2}\right).$$

Variable $p_{1}$ is monotonically increasing from zero to the equilibrium frequency $p_{20}$. The two most important parameters are $s_{1}$ and r. Larger values of $\left|s_{1}\right|$ and less linkage between loci (i.e., larger r values) lead to faster increase of $p_{1}$.

Next, we calculate (for a fixed time point at the end of the selective phase) the effect of strong selection at the second locus on the frequency of allele *A* and on heterozygosity at the first locus. Let p denote the frequency of *A* and $H=2p\left(1‐p\right)$ heterozygosity. Thus,

(35) $$p=p_{1}x+p_{2}\left(1‐x\right).$$

At the end of the selective phase at time $\hat{\tau }=‐\frac{2}{s_{2}}\ln\left(x_{0}\right)$ when x has reached the frequency $1‐x_{0}$ we have

(36) $$p\left(\hat{\tau }\right)\approx p_{1}\left(\hat{\tau }\right)\left(1‐x_{0}\right)\approx p_{20}\left[1‐x_{0}^{‐2s_{1}/s_{2}}\left(1‐\frac{r}{\mu_{A}+r}\left(1‐x_{0}^{2\left(\mu_{A}+r\right)/s_{2}}\right)\right)\right].$$

For the case in which the polymorphism at the first locus is neutral, this expression agrees with previous results (Maynard Smith & Haigh, 1974; Stephan et al., 1992; Wiehe, 1995).

Based on Eq. (36), we can immediately predict the effect of strong selection at the second locus on heterozygosity at the first locus. Heterozygosity is an average over two events, as allele *B* arises with probability $p_{20}$ on an *A*‐carrying chromosome or with probability $1‐p_{20}$ on an *a*‐chromosome (Kaplan et al., 1989; Stephan et al., 1992). However, since in a mutation‐selection balance $p_{20}$ is small, we may neglect the first event and obtain

(37) $$H\left(\hat{\tau }\right)\approx 2\left(1‐p_{20}\right)p\left(\hat{\tau }\right)\left(1‐p\left(\hat{\tau }\right)\right)\approx 2\left(1‐p_{20}\right)p\left(\hat{\tau }\right).$$

Therefore, the ratio of heterozygosity after the sweep (at $\tau =\hat{\tau })$ to heterozygosity before the sweep at τ=0 is given by

(38) $$\frac{H\left(\hat{\tau }\right)}{H\left(0\right)}\approx 1‐x_{0}^{‐2s_{1}/s_{2}}\left(1‐\frac{r}{\mu_{A}+r}\left(1‐x_{0}^{2\left(\mu_{A}+r\right)/s_{2}}\right)\right).$$

Thus, heterozygosity at the first locus is reduced after a sweep caused by strong selection at a linked second locus. The relative reduction of variation, i.e., the hitchhiking effect, is, however, less pronounced than in the case of a neutral polymorphism at the first locus.

## 4 STOCHASTIC ANALYSIS OF INITIAL PHASE

As mentioned above, the dynamics of the beneficial allele at very low frequency ($x\leq x_{0}$) cannot be treated deterministically. Instead, we will use a diffusion approach. Assuming $s_{2}>0,\mu_{B}>0,r=0,\mu_{A}={\bar{\mu }}_{A}=0,\;\text{and}\;s_{1}=0$, we will derive an appropriate diffusion equation for a diploid population of constant size N and then calculate the first and second moments of this diffusion. We will first consider the frequency process of the beneficial allele and put z=x as diffusion variable. From Eq. (8), we find the drift coefficient as

(39) $$a\left(z\right)\approx \alpha z+\frac{1}{2}\theta ,$$

where $\alpha =2Ns_{2}$ and $\theta =4N\mu_{B}.$ The selection term is linear in z, as the frequency of the beneficial allele in the initial phase is very low. In the initial phase, the diffusion coefficient is also linear in z; i.e.,

(40) $$b\left(z\right)\approx z.$$

Thus, we have the following Kolmogorov forward equation describing a one‐dimensional diffusion in the initial phase (Ewens, 2004, Chapter 4)

(41) $$\frac{\partial f\left(z,t\right)}{\partial t}=‐\frac{\partial }{\partial z}\left(a\left(z\right)f\left(z,t\right)\right)+\frac{1}{2}\frac{\partial^{2}}{\partial z^{2}}\left(b\left(z\right)f\left(z,t\right)\right).$$

The probability density function f of this equation has to satisfy some assumptions: The initial frequency of z is 0 at time t=0, which denotes the onset of selection; furthermore, f is normalized to 1 and the boundary conditions are such that for larger z values both $f\left(z,t\right)$ and $\frac{\partial }{\partial z}f\left(z,t\right)$ converge to 0 (i.e., $f\left(1,t\right)=\frac{\partial }{\partial z}f\left(1,t\right)=0$). Furthermore, in this section time is scaled in units of 2N.

We do not aim at finding an explicit solution of Eq. (41), but consider only the two lowest‐order moments defined as

(42) $$m_{i}=\int_{0}^{1}z^{i}f\left(z,t\right)dz\;\text{for}\;i=1,2.$$

This procedure leads to the following ODEs for the moments (see Appendix B):

(43) $${\dot{m}}_{1}\approx \frac{1}{2}\theta +\alpha m_{1}$$

and

(44) $${\dot{m}}_{2}\approx \left(1+\theta \right)m_{1}+2\alpha m_{2}.$$

Thus, we get a coupled system of ODEs. Note, however, that—contrary to many other applications of this approach—the moment expansion breaks up (as the drift and diffusion coefficients do not contain the variable z in quadratic or higher‐order forms). The ODE for the first moment corresponds to Eq. (8) of the deterministic system.

The solutions of these linear ODEs of first order can be easily obtained as

(45) $$m_{1}\approx \frac{\theta }{2\alpha }\left(e^{\alpha t}‐1\right)$$

and

(46) $$m_{2}\approx \left(1+\frac{1}{\theta }\right)m_{1}^{2}.$$

Thus, the variance of x is given by

(47) $$m_{2}‐m_{1}^{2}\approx \frac{1}{\theta }m_{1}^{2}.$$

Similarly, we may consider a one‐dimensional diffusion equation for variable $z=x_{1}$ describing the frequency process of gamete *AB*. It follows from ODE (1) that in the initial phase the drift coefficient is given by

(48) $$a\left(z\right)\approx \alpha z+\frac{1}{2}\theta \sigma \left(t\right)$$

and the diffusion coefficient by Eq. (40); $\sigma \left(t\right)$ is the solution of ODE

(49) $${\dot{x}}_{3}=‐\left(\mu_{B}+s_{2}x\right)x_{3}$$

derived from Eq. (3) and given by

(50) $$\sigma \left(t\right)=x_{3}^{*}\exp\left(‐\frac{\theta }{2\alpha }\left(e^{\alpha t}‐1\right)\right),$$

where $x_{3}^{*}$ is the frequency of allele *A* at the onset of selection. The ODEs of the moments may be obtained by the same procedure as outlined in the Appendix B. We get

(51) $${\dot{m}}_{1}\approx \frac{1}{2}\theta \sigma \left(t\right)+\alpha m_{1}$$

and

(52) $${\dot{m}}_{2}\approx \left(1+\theta \sigma \left(t\right)\right)m_{1}+2\alpha m_{2}.$$

Using Eq. (50), ODE (51) can be formally integrated

(53) $$m_{1}\approx \frac{1}{2}\theta x_{3}^{*}e^{\alpha t}\int_{0}^{t}\exp\left(‐\frac{\theta }{2\alpha }\left(e^{\alpha t^{\prime }}‐1\right)‐\alpha t^{\prime }\right)dt^{\prime }.$$

The integral cannot be explicitly evaluated for all values of t. However, close inspection shows that for the biologically relevant parameter range (α>100,θ>0.005), the integrand in Eq. (53) can be approximated by $e^{‐\alpha t^{\prime }}\;\text{for}\;t^{\prime }\leq 2{\bar{t}}_{0},$ where ${\bar{t}}_{0}$ is defined below. This analysis takes the variance of the x diffusion (Eq. (47)) into account. Using this approximation, we get

(54) $$m_{1}\approx \frac{\theta }{2\alpha }x_{3}^{*}\left(e^{\alpha t}‐1\right).$$

Here, the mean time ${\bar{t}}_{0}$ until the beneficial allele *B* reaches the threshold frequency $x=x_{0}$ under the influence of drift, directional selection, and mutation (starting from frequency 0) is given by

(55) $${\bar{t}}_{0}\approx \frac{1}{\alpha }\ln\left(1+\frac{2\alpha }{\theta }x_{0}\right).$$

Using the same approximation as in the derivation of Eq. (54), we obtain for the second moment of the $x_{1}$ process

(56) $$m_{2}\approx \left(1+\frac{1}{\theta x_{3}^{*}}\right)m_{1}^{2}.$$

Finally, we are able to determine the initial conditions for the deterministic phase. Eqs. (54) and (55) allow us to calculate the value of $p_{1}=\frac{x_{1}}{x}$ at time $t_{0}$, i.e., at the beginning of the deterministic phase. We find $p_{1}\left(t_{0}\right)=p_{10}\approx x_{3}^{*}$. However, since the variances of the diffusions x and $x_{1},$ for which we got simple analytical formulas (see Eqs. (47) and (56)), are relatively large, $p_{1}$ may not be well predicted by the first moments of x and $x_{1}$ at time $t_{0}.$ In contrast, the value of $p_{2}\left(t_{0}\right),$ which is defined as a ratio of two relatively large quantities (≥0.5) shortly after the onset of selection, is evidently better predicted. Using Eq. (50), we get $p_{2}\left(t_{0}\right)=\frac{x_{3}\left(t_{0}\right)}{1‐x_{0}}=p_{20}\approx x_{3}^{*}$. Thus, $p_{2}\left(t_{0}\right)$ is close to its value $x_{3}^{*}$ at t=0, which is expected.

On the other hand, if indeed $p_{10}=p_{20},$ as expected, we get the interesting result that at the time of fixation $x_{1}=p_{10}$ (see Eqs. (12) and 14)). That means that the ratio of the frequency of *AB* gametes to the frequency of *B* alleles is constant during the selective phase from time $t_{0}$ to fixation. The effect of mutation during this phase is therefore negligible. In other words, the competition between mutation and selection (and drift) is expected to occur exclusively during the initial phase.

## 5 DISCUSSION

We extended the classical two‐locus two‐allele hitchhiking model of Maynard Smith and Haigh (1974) by allowing mutation and selection at both loci. Besides strong positive directional selection at the selected locus, we allow for weak purifying selection at the linked locus. We show that the hitchhiking effect expressed by a reduction of variation is weaker when the polymorphism at the linked locus is in a mutation‐selection balance rather than neutral.

Furthermore, introducing mutation from the wild type to the strongly beneficial allele may lead to the occurrence of multiple‐origin soft sweeps in the extended hitchhiking model. We identified a new parameter, $\frac{\mu_{B}}{s_{2}},$ determining the occurrence of this type of sweep. The proportion of soft sweeps (conditional on sweeps are occurring) is predicted to increase with increasing values of $\frac{\mu_{B}}{s_{2}}$. This result may be compared with the simulations of Feder et al. (2021) who proposed a simulation model to explain certain features of HIV evolution. In their Figure 3C, they show for a fixed beneficial mutation rate that increasing the selection coefficient leads to a strong reduction of the percentage of multiple‐origin soft sweeps, which is in qualitative agreement with our analysis.

We also analyzed the initial phase when—after the onset of strong positive selection—the frequency of the beneficial allele is very small (≪1). Based on diffusion theory, we calculated the first and second moments of the frequencies x(benefical allele) and $x_{1}$(gamete *AB*). This helped us to quantify the initial conditions of the deterministic ODEs, which we needed to analyze our extended deterministic hitchhiking model. Our approach is based on the same biological assumptions as that of Martin and Lambert (2015) who analyzed the frequency process of the beneficial allele of the original hitchhiking model (i.e., without mutation at the selected locus). They used the (linear) Feller diffusion process for which more short‐term results can be obtained explicitly than for a Wright‐Fisher diffusion.

In the theoretical analysis of selective sweeps, several questions have not been satisfactorily addressed (Stephan, 2019). A major one concerns the traffic model. Although this model has been proposed 25 years ago (Barton, 1995; Kirby & Stephan, 1996), not much progress has been made in analyzing it. Most analyses still assume that selective sweeps along the genome occur sequentially, without interfering with each other. However, imagine a model with two partially linked loci at which beneficial mutations may enter a population independently. An interesting scenario arises when a second mutation *B* with higher fitness occurs, while the first one (*A*) is on its way to fixation. If *A* and *B* can recombine at some rate, there is a chance that the double beneficial mutant *AB* forms and eventually fixes. Basic questions such as the fixation probability of *AB* and its fixation time have been addressed in a series of mathematical papers (Bossert & Pfaffelhuber, 2018; Cuthbertson et al., 2012; Yu & Etheridge, 2010). However, the pattern of variation in genetic data for such a model of competing sweeps is largely unknown.

The only report on patterns of variation in recombining genomic regions has been published by Chevin et al. (2008). They modeled the case of two partially linked loci with positive directional selection at both of them and one neutral locus for an infinitely large population using ordinary differential equations. Solving these equations numerically, they found that the hitchhiking effect is weaker in this model than for a single sweep of comparable selection strength. Furthermore, the interference of both sweeps may lead to an excess of intermediate‐frequency variants in the genomic region between the selected sites, a signature that may be falsely interpreted as a sign of balancing selection. More work is needed to understand such a model.

Similarly, selective sweep models from the quantitative genetics literature have been relatively neglected by the population genetics community, such as the work of Santiago and Caballero (1995, 1998). These authors developed a quantitative genetic theory of effective population size and polymorphism of linked neutral loci in populations under directional selection and continuous mutation pressure. Interestingly, they were able to apply the principles of their theory to the recurrent hitchhiking case by considering a steady input of weakly beneficial mutations instead of rare, strongly favorable ones, as is usually assumed in the model of recurrent selective sweeps (Kaplan et al., 1989; Wiehe & Stephan, 1993).

## CONFLICT OF INTEREST

None declared.

## AUTHOR CONTRIBUTION

**Wolfgang Stephan:** Conceptualization (lead); Formal analysis (lead); Funding acquisition (lead); Writing‐review & editing (lead).

## Data Availability

No empirical data were collected, and no computer code was generated or used.

## APPENDIX A

### Condition for the occurrence of soft sweeps may be violated

In our model a soft sweep can be detected conditional on a sweep has occurred (i.e. x=1), if the frequencies $x_{1}$ and $x_{2}$ of the gametes *AB* and *aB* are both larger than a threshold value ε<0.5. For $x_{1}$ this condition, however, may be violated. Using Eq. (14) and inserting x=1, we find that $x_{1}<\varepsilon$ if

(A1)
$$\mu_{B}<s_{2}x_{0}I\left(p_{10},p_{20}\right),$$

where

(A2)
$$I\left(p_{10},p_{20}\right)=\frac{\varepsilon ‐p_{10}}{p_{20}\left(1‐x_{0}\right)+x_{0}p_{10}‐\varepsilon }\approx \frac{\varepsilon ‐p_{10}}{p_{20}‐\varepsilon }$$

is a positive function (i.e. $p_{10}<\varepsilon ).$ As shown in the second to last paragraph of section 4, $p_{20}$ is approximately given by the frequency $x_{3}^{*}$ of the major allele *A* at the onset of selection, while $p_{10}<\varepsilon$ may occur due to the large variances of x and $x_{1}$ in the initial phase.

## APPENDIX B

### Derivation of the moments

Using the definition of the moments (Eq. (42)) and integrating by parts, we get

(B1)
$$\int_{0}^{1}z^{i}\frac{\partial }{\partial z}\left(\left(\alpha z+\frac{1}{2}\theta \right)f\left(z,t\right)\right)dz$$

(B2)
$$=\left[z^{i}\right.{\left.\left(\alpha z+\frac{1}{2}\theta \right)f\left(z,t\right)\right]}_{0}^{1}$$

(B3)
$$‐\int_{0}^{1}iz^{i‐1}\left(\alpha z+\frac{1}{2}\theta \right)f\left(z,t\right)dz.$$

For both i=1and2 the term (B2) vanishes because of the boundary condition $f\left(1,t\right)=0.$ For i=1 integral (B3) yields $\frac{1}{2}\theta +\alpha m_{1}$ (without the – sign in front of the integral), whereas for i=2 we find $\theta m_{1}+2\alpha m_{2}$.

Similarly, we obtain the contribution of the diffusion part of Eq. (41) as

$$\int_{0}^{1}z^{i}\frac{\partial^{2}}{\partial z^{2}}\left(zf\left(z,t\right)\right)dz$$

$$=\left[z^{i}\right.{\left.\frac{\partial }{\partial z}\left(zf\left(z,t\right)\right)\right]}_{0}^{1}$$

$$‐\int_{0}^{1}iz^{i‐1}\frac{\partial }{\partial z}\left(zf\left(z,t\right)\right)dz$$

(B4)
$$=\left[z^{i}\right.f\left(z,t\right)+{\left.z^{i+1}\frac{\partial }{\partial z}f\left(z,t\right)\right]}_{0}^{1}$$

(B5)
$$‐\int_{0}^{1}iz^{i‐1}\frac{\partial }{\partial z}\left(zf\left(z,t\right)\right)dz.$$

Here, the term (B4) vanishes for both i=1and2 because of the boundary conditions $f\left(1,t\right)=\frac{\partial }{\partial z}f\left(1,t\right)=0.$ The integral (B5) vanishes for i=1 because of $f\left(1,t\right)=0.$ For i=2 a similar calculation as for the integral (B1) shows that integral (B5) is equal to 2$m_{1}.$

Collecting the non‐zero terms for i=1 leads to the ODE for the first moment shown in Eq. (43). Note that the minus signs in front of integral (B3) and in front of the drift term of the diffusion equation cancel each other. Similarly, collecting the terms for i=2 and multiplying them by $\frac{1}{2}$ leads to the ODE for the second moment (Eq. (44)).
